# Selective RNAVersus DNA G-Quadruplex Targeting by In Situ Click Chemistry**

**DOI:** 10.1002/anie.201206281

**Published:** 2012-10-04

**Authors:** Marco Di Antonio, Giulia Biffi, Angelica Mariani, Eun-Ang Raiber, Raphaël Rodriguez, Shankar Balasubramanian

**Affiliations:** University of Cambridge, Department of ChemistryLensfield Road, Cambridge, CB2 1EW (UK); * Cambridge Research Institute, Cancer Research UK, Li Ka Shing CentreCambridge, CB2 0RE (UK)

**Keywords:** click chemistry, DNA, G-quadruplexes, RNA, telomeres

Click chemistry has been broadly defined as chemical reactions capable of promoting the formation of stable covalent adducts from two distinct reactive moieties in a mild and quantitative manner with an appreciable tolerance to water.^1^ These stringent requirements have rendered the development of click reactions particularly challenging.^2^ More recently, the term click chemistry has referred to the 1,3-dipolar cycloaddition of alkynes and azides that is promoted at room temperature by various copper- and ruthenium-based catalysts.^3^ This powerful reaction has found broad applications that span from material science to cell biology.^4^ Copper-free variants involving reactive cyclooctyne derivatives have been widely investigated to enable its use in cellulo, in particular for the labeling of biomolecules.^5^ Conversely, the correct orbital alignment of terminal alkynes and azides necessary for the cycloaddition to take place is well known to increase the negative values of Δ*S*^≠^, thus making the noncatalyzed reaction rather slow in a biologically relevant environment. This feature, initially perceived as a limitation, was exploited by Finn and Sharpless in their concept of “in situ” click chemistry, which relies on the specific interactions between the reactants and a given biological target to enable the optimal orientation of the substrates for the reaction to occur at room temperature.^6^ In situ click chemistry has led to the discovery of several enzyme inhibitors with low nanomolar *K*_d_ values.^7^ There is as yet no report on such an approach to generate small molecules that selectively target a defined secondary structure of a nucleic acid.

Herein, we demonstrate the value of in situ click chemistry approaches to identify potent and selective small molecules that bind G-quadruplexes (G4). We have selected the human telomeric DNA as a suitable target because of its relevance to cancer biology.^8^ Telomeres play a crucial cellular function by protecting chromosomes from shortening after several rounds of replication, thereby sustaining the stability of the genome. This region of chromosomes is composed of tandem repeats of guanine tracts (TTAGGG)_*n*_ that are prone to fold into polymorphic G4 structures (H-Telo).^9^ It has previously been shown that small molecules capable of interacting with H-Telo have the ability to activate a DNA damage response at telomeres and to trigger senescence and apoptosis.^10^ Therefore, we have explored the ability of H-Telo to act as the reaction vessel for the 1,3-dipolar cycloaddition to generate potent telomere-targeting small molecules.

Two G4 binding substrates **1** and **2** (Figure 1 A) containing an alkyne substituent were used as the starting materials. Alkynes **1** and **2** were derived from the known G4-binding small molecule pyridostatin (PDS), but lacked a polar function on the central pyridine core, thereby leaving room to modulate the binding properties of the substrate through a click reaction with a series of azides.^11^ Azides **3**–**8**, which comprise positively and negatively charged atoms, aromatic substrates, and a neutral sugar, were used to cover several types of interaction modes, including π stacking as well as electrostatic and hydrogen bonding, with the aim of maximizing the chances of identifying a strong DNA G4 interacting partner.

**Figure 1:**
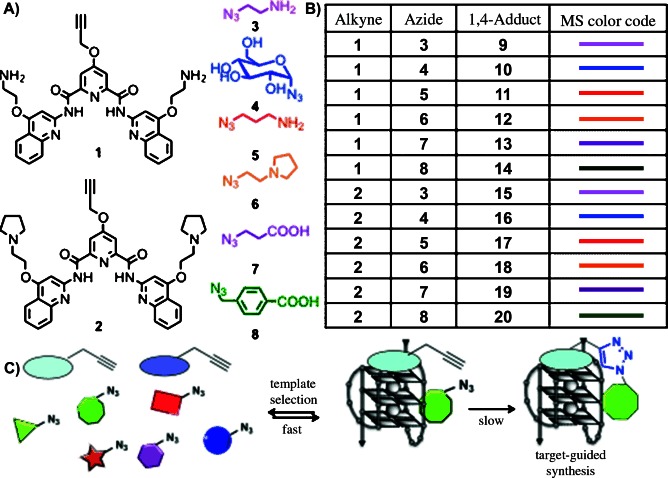
A) Molecular structure of alkyne and azide building blocks; B) adducts generated by treating **1** and **2** with **3**–**8**; C) in situ synthesis of triazoles catalyzed by H-Telo.

We anticipated that the presence of the DNA target in the mixture would catalyze the formation of potent adducts (Figure 1 B).^12^ In a typical experiment, alkynes **1** and **2** (25 μm), azides **3**–**8** (1 mm), Tris⋅HCl (10 mm, pH 7.4; Tris=tris(hydroxymethyl)aminomethane), and KCl (250 mm) containing buffer, with and without telomeric DNA G4 (25 μm) were mixed together. A 40-fold excess of each individual azide was used to alleviate any bias imposed by changes in the concentration of the azides selected during the course of the reaction.

To ensure that products of the click reaction were the result of specific interactions between the reactants and the structured DNA G4, we independently performed a reaction either in the presence of a double-stranded DNA (ds-DNA) control (25 μm) or with the telomeric oligonucleotides pre-annealed in a lithium-containing buffer, which is known to prevent G4 formation.^13^ Each solution was stirred at room temperature for six days, before trifluoroacetic acid (TFA) was added to denature the DNA (20 % aq). The mixture was monitored by LC-MS to simultaneously identify the nature and quantify the respective amounts of each product from a reaction that could, in principle, contain up to 24 cycloadducts, including 1,4- and 1,5-regioisomers (Figure 1 C). The mass spectrometer was programmed to independently detect the mass of each possible adduct and alkyne substrate by using a single ion monitoring protocol, thus enabling the analysis of the complex reaction mixture with high resolution. Interestingly, we observed the formation of the single 1,4-adduct **10**, which results from the cycloaddition of **1** and **4** in the presence of H-Telo (Figure 2), whereas no adduct could be detected when reactions were either conducted in the absence of DNA or in the presence of ds-DNA.^14^ In addition, no adduct was observed when the telomeric oligonucleotide was pre-annealed in lithium-containing buffer prior to its use, thus demonstrating that the G4 structure is required for the 1,3-dipolar cycloaddition to occur under the mild reaction conditions used. These data suggest that the dynamic and reversible assembly of alkynes and azides with the G4 catalyst is a fast process that allows the system to select the most potent building blocks prior to slowly reacting with one another to generate the adduct, a procedure evocative of dynamic combinatorial processes.^15^ It is noteworthy that the neutral sugar-containing azide was selected at the expense of positively charged azides, a rather counterintuitive outcome considering previously reported data recorded for the potent G4 ligand PDS and other amine-containing G4-binding small molecules.^16^ Additionally, no adduct could be detected when azide **4** was removed from the reaction mixture. This finding indicates that specific interactions occur between **1**, **4**, and the DNA catalyst, while the other azides, once bound to H-Telo, may be held remote from the alkyne substrates in a way that does not favor the cycloaddition under these conditions. Overall, these results show that **1** is a better ligand than **2** and that adduct **10** exhibits enhanced interacting capabilities compared to the other possible adducts and starting materials.

**Figure 2:**
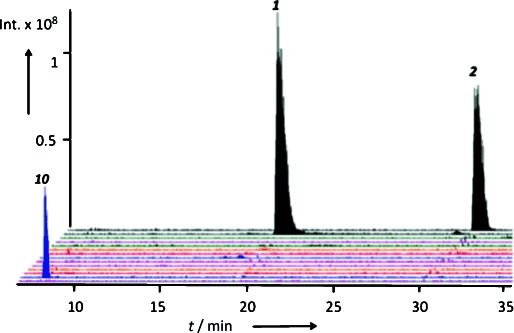
Chromatogram tuned on 14 mass channels of alkynes **1** and **2** and adducts **9**–**20** obtained from the reaction carried out in the presence of H-Telo.

The clear effect of H-Telo on the 1,3-dipolar cycloaddition prompted us to investigate whether the presence of copper could compensate for the poor kinetics, whilst preserving a good selectivity. Thus, a similar reaction containing all the building blocks and an excess of a copper catalyst was performed. Figure 3 A depicts a stochastic distribution of the reaction products that reflects the initial concentration and intrinsic reactivity of each individual starting material. Under these reaction conditions, incubation at room temperature for 3 h was sufficient for the reaction to reach completion. In a separate experiment where a substoichiometric amount of H-Telo (0.05 mol equiv) was added to the mixture containing the copper catalyst, we observed that the distribution of products was biased towards sugar-containing adducts **10** and **16** at the expense of the others identified in the presence of only copper (Figure 3 B). This indicates that, to some extent, the DNA G4 overrides the lack of specificity imposed by the kinetically favored copper-catalyzed reaction.

**Figure 3:**
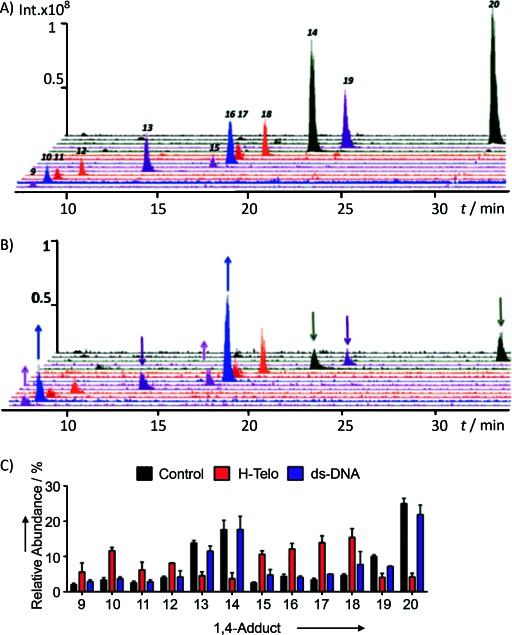
Chromatogram tuned on the 14 mass channels of alkynes **1** and **2** and adducts **9**–**20** obtained from the reaction carried out in the presence of A) a Cu catalyst and B) a Cu/H-Telo catalyst. C) Histogram showing the relative abundance of each product obtained in copper- (control), Cu/H-Telo-, and Cu/ds-DNA-catalyzed reactions. Data were plotted as the surface area measured under each individual chromatogram signal.

Indeed, these data demonstrate that while copper controls the reaction kinetics, the selectivity is still imposed by the DNA template, thus suggesting a cooperative behavior of the H-Telo and copper catalysts in a manner that is reminiscent of DNAzymes.^17^

Furthermore, this result suggests that **10** does not poison the DNA catalyst, despite a higher shift in the melting temperature of H-Telo induced by **10** compared to **1**, according to fluorescence resonance energy transfer (FRET) melting experiments, thereby showing that **10** is a better stabilizer than **1** (see Table 1).^18^, ^19^

**Table 1:** FRET melting values for H-Telo and ds-DNA in K

Compound (1 μm)	Δ*T*_m_ (H-Telo)	Δ*T*_m_ (ds-DNA)
**1**	16.0±0.1	0.0±0.1
**2**	12.8±0.2	0.0±0.1
**9**	21.1±0.2	0.0±0.1
**10**	30.0±0.1	0.0±0.1
**11**	18.8±0.6	0.0±0.1
**12**	22.5±0.7	0.0±0.1
**13**	11.2±0.1	0.0±0.1
**14**	6.3±0.4	0.0±0.1
**15**	19.1±0.3	0.0±0.1
**16**	28.2±1.1	0.1±0.1
**17**	17.2±0.3	0.0±0.1
**18**	20.1±0.2	0.0±0.1
**19**	9.1±0.5	0.0±0.1
**20**	5.9±0.3	0.0±0.1
PDS	35.4±1.2	0.0±0.1

The favorable turnover observed may be the result of the well-known folding/unfolding dynamic properties of G4 nucleic acids.^13^, ^20^ In contrast, the production of negatively charged adducts, which are known to be weaker binders because of electrostatic repulsions, was poor compared to those of other adducts, and significantly lower than their respective amounts obtained from experiments conducted in the absence of H-Telo. In line with the data obtained from copper-free experiments, the presence of ds-DNA in copper-catalyzed reactions had negligible effect on the product distribution, as shown in Figure 3 C (see also the Supporting Information). To provide a rationale underlying the catalytic effect of H-Telo we analyzed the stabilization properties of each individual starting material and adduct by FRET melting experiments. Table 1 displays the increase in the melting temperatures of H-Telo (Δ*T*_m_) in the presence of each compound, measured independently. These data show a general trend in which adducts derived from **1** generally exhibit slightly better stabilizing properties than the analogues derived from **2**. Furthermore, we observed that the stabilization properties of each adduct followed the general trend of neutral (sugar)>positively charged (amines)>negatively charged (carboxylic acids), with Δ*T*_m_ values ranging from 30 K for sugars to 20 K for amines and 6 K for carboxylic acids. Finally, adducts were generally more potent than the starting materials **1** and **2**, with the exception of negatively charged adducts, which were even weaker stabilizers. This general trend is in strong agreement with the DNA-guided synthesis profiles observed in the copper-catalyzed reaction, thus supporting a model whereby the DNA G4 catalyzes the formation of the most potent interacting small molecules (i.e sugar and amino analogues).

Shelterin is a six-protein complex that binds to telomeric DNA to protect telomeres from the DNA damage response machinery and to prevent chromosomes from shortening.^21^ This complex is transiently dissociated from DNA during the dynamic processes of replication and transcription, hence enabling small molecules to interact with the DNA and to compete out shelterin components. Therefore, we investigated the ability of diverse adducts to target telomeric DNA by measuring the amount of the shelterin component telomeric repeat-binding factor 1 (TRF1) at telomeres by using fluorescence microscopy. Human MRC5-SV40 fibroblasts were incubated with various analogues at 2 μm for 24 h and the TRF1 protein was visualized and quantified by using an anti-TRF1 antibody. Remarkably, the number of TRF1 foci per cell strongly decreased in a dose-dependent manner upon treatment with **10** compared to untreated cells, with an IC_50_ value of (1.3±0.3) μm, whereas a negligible effect was observed for the negatively charged control **20** and precursors **1** and **2** (Figure 4). This result is in strong agreement with the copper-free in situ synthesis of the ligand, where only the selected molecule **10** is able to compete out the shelterin component from telomeres. Consistent with this, negligible TRF1 displacement was observed for other adducts such as **9** or carboxypyridostatin (**13**) under the same conditions (see the Supporting Information), thus validating the in situ approach for the discovery of structure-selective small molecules. It is noteworthy that the parent molecule PDS induced the highest increase in the melting temperature of H-Telo (Table 1), but did not induce significant phenotypic changes at telomeres under similar conditions. Overall, these results demonstrate that in situ click chemistry is an accurate and unbiased method to generate and evaluate biologically active small molecules in a single step and can be more powerful than preexisting methods based on rational design. This concept may be exemplified by the observation that PDS was predicted to be the most potent hit for this particular target according to FRET melting experiments, with a Δ*T*_m_ value of 35 K compared to 30 K for **10**, while in situ click selection generated the slightly weaker stabilizer **10** that is a more potent TRF1 competitor in cellular experiments.

**Figure 4:**
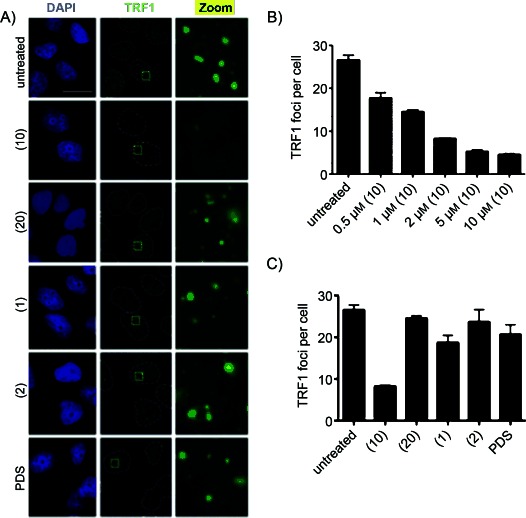
A) Fluorescent microscopy images of untreated MRC5-SV40 cells or treated with 2 μm compound for 24 h. Cells were stained with 4,6-diamidino-2-phenylindole (DAPI) and anti-TRF1 antibody; scale bar: 20 μm, zoom images are 25X magnified. B) Dose-response readout based on the number of TRF1 foci per cell upon treatment with **10** for 24 h. C) Number of TRF1 foci per cell upon treatment with various adducts and PDS (2 μm, 24 h).

Finally, we sought to explore whether our method would enable the identification of small molecules with the ability to selectively interact with RNA G4 structures but not their DNA counterparts. To this end, the telomeric repeat-containing RNA (TERRA), known to form a stable G4 structure in vitro, was used as a catalyst.^22^ Analysis of the reaction mixture conducted in the presence of Cu/TERRA catalysts revealed significant differences in the respective abundance of the products compared to the reaction conducted in the presence of the Cu/H-Telo catalysts (Figure 5 A; see also the Supporting Information).

**Figure 5:**
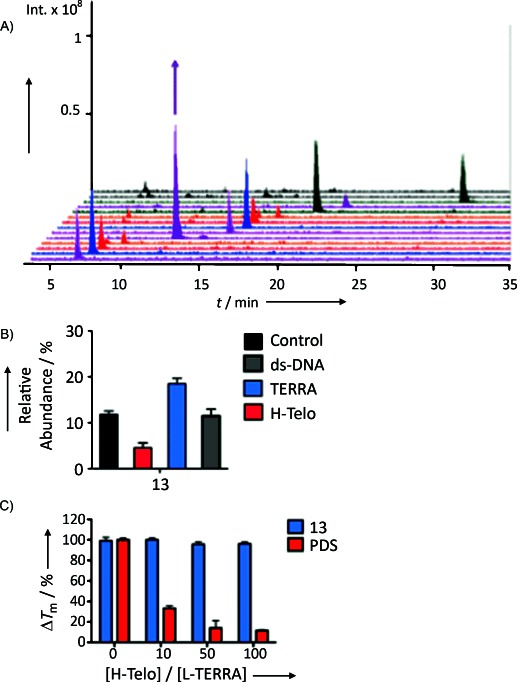
A) Chromatogram tuned on the 14 mass channels of alkynes **1** and **2** and adducts **9**–**20** obtained from the reaction carried out in the presence of Cu/TERRA catalysts. B) Histogram showing the relative abundance of **13** obtained from Cu- (control), Cu/H-Telo-, Cu-TERRA-, and Cu/ds-DNA-catalyzed reactions. C) Histogram showing the shift in the melting temperature of doubly labeled TERRA in the presence of **13** or PDS (2 μm) and increasing amounts of unlabeled H-Telo competitor.

Strikingly, the negatively charged adduct **13** was the most prevalent product of the reaction conducted in the presence of Cu/TERRA, whereas its abundance was decreased in the presence of H-Telo (Figures 3 and 5 A,B). In contrast, the abundance of the other negatively charged adduct **14** containing an aromatic moiety was lower in the reaction conducted in the presence of Cu/TERRA compared to that in the copper-catalyzed reaction. This finding indicates that very selective interactions are involved between the RNA catalyst and substrates **1** and **7**. It also challenges the idea that selective nucleic acid targeting is restricted to neutral and positively charged small molecules.^23^ This difference in chemical reactivity may be the result of hydrogen bonding of the carboxylate moiety of **7** and the 2′-OH group of the RNA substrate, which is absent in the corresponding DNA template. This notion is further supported by the fact that the RNA G4 unit present in the 5′-UTR of *NRAS* exhibited a similar catalytic effect as TERRA, with isolation of **13** as the major product of the reaction.^24^ FRET melting experiments confirmed that **13** is a better TERRA stabilizer than the alkyne precursor **1** and product **14**, with Δ*T*_m_ values of (20.7±0.1) K, (15.1±0.2) K, and (6.4±0.2) K at 1 μm compound, respectively, whereas PDS was the most potent stabilizer with a Δ*T*_m_ value of (22.2±0.1) K (see the Supporting Information). In competition experiments where TERRA was doubly labeled (L-TERRA) and an excess of the unlabeled H-Telo target competitor was added, we observed that the ability of **13** to stabilize TERRA was not affected by the presence of up to 100 mol equiv DNA competitor, whereas the ability of PDS to stabilize TERRA decreased by (19.5±0.4) K upon titration with the H-Telo target (Figure 5 C). This result demonstrates that while PDS is a good generic RNA and DNA G4-interacting small molecule, the negatively charged adduct **13** is a selective RNA G4-interacting compound.^25^

We previously reported the existence of other G4 structures in the coding region of human genes and showed that PDS was a potent generic G4-interacting small molecule, somewhat lacking the ability to trigger a strong response at telomeres after a short treatment period.^26^ The cellular phenotype induced by PDS, that is the induction of DNA damage at specific human genomic site containing G4 clusters, may to some extent be linked to RNA G4 structures, in particular with respect to RNA splicing during transcription and telomere maintenance. For example, it has been proposed that TERRA acts as a negative regulator of telomerase by competing with the DNA substrate.^27^ The unique ability of carboxypyridostatin to selectively stabilize the TERRA G4 structure but not H-Telo provides the means to elucidate the existence, and decipher the biological function, of RNA selectively from DNA G4 structures in human cells. This study demonstrates that unbiased approaches based on in situ click chemistry are suitable to enhance the ability of small molecules to interact with a given nucleic acid structure. Such a strategy paves the way towards structure-selective nucleic acid targeting, which is notoriously difficult to achieve.

## Supplementary material

Detailed facts of importance to specialist readers are published as ”Supporting Information”. Such documents are peer-reviewed, but not copy-edited or typeset. They are made available as submitted by the authors.
