# Supplementary material for: Selective RNAVersus DNA G-Quadruplex Targeting by In Situ Click Chemistry
Source: Angew Chem Int Ed Engl. 2012 Oct 4;51(44):11073–8. doi: 10.1002/anie.201206281 (PMC3652031; doi:10.1002/anie.201206281)
Supplement: Supplementary file 1 [file anie0051-11073-SD1.pdf]

Supporting Information

© Wiley-VCH 2012

69451 Weinheim, Germany

Selective RNA Versus DNA G-Quadruplex Targeting by In Situ Click Chemistry**

Marco Di Antonio, Giulia Biffi, Angelica Mariani, Eun-Ang Raiber, Raphaël Rodriguez, and Shankar Balasubramanian**

anie_201206281_sm_miscellaneous_information.pdf

Contents

**1.**	**Synthesis and general experimental procedures**	**S2**
**2.**	**NMR and HRMS spectral data**	**S10**
**3.**	**Molecular structures of compounds 9-20**	**S14**
**4.**	**Click chemistry procedures**	**S15**
**5.**	**HPLC method and chromatograms**	**S16**
**6.**	**Products distribution for Cu catalyzed click reactions**	**S17**
**7.**	**FRET-melting curves**	**S17**
**8.**	**TRF1 immunostaining**	**S18**
**9.**	**References**	**S20**

1. Synthesis and general experimental procedures

All solvents and reagents were purified by standard techniques reported in Armarego, W. L. F., Chai, C. L. L., Purification of Laboratory Chemicals, 5th edition, Elsevier, 2003; or used as supplied from commercial sources (Sigma-Aldrich Corporation® unless stated otherwise). NMR spectra were acquired on Bruker® DRX-400, Bruker® DPX-400 and DRX-500 instruments using deuterated solvents as detailed and at ambient probe temperature (300 K). Notation for the ^{1}H NMR spectral splitting patterns includes: singlet (*s*), doublet (*d*), triplet (*t*), broad (*br*) and multiplet/overlapping peaks (*m*). Signals are quoted as δ values in ppm, coupling constants (*J*), are quoted in Hertz and approximated to the nearest 0.5. Data analysis for the nuclear magnetic resonance (NMR) spectra was performed using TopSpin® software. Mass spectra were recorded on a Micromass® Q-Tof (ESI) spectrometer. Thin layer chromatography (TLC) was performed on Merck Kieselgel 60 F254 plates, and spots were visualized under UV light. Flash chromatography (FC) was performed using Merck Kieselgel 60 at room temperature under a positive pressure of nitrogen using previously distilled solvents. High performance liquid chromatography (HPLC) purification was carried out on all final compounds by using a Varian Pursuit C18, 5 *μ* column (250 × 21.2 mm) and a gradient elution with H_2O / acetonitrile (MeCN) containing 0.1% TFA at a flow rate of 12.0 ml/ min. LC/MS analysis has been performed with all final compounds, which had a purity of ≥95%.

FRET-melting studies. 100 μM stock solutions of oligonucleotides were prepared in molecular biology grade DNase-free water. Further dilutions were carried out in 60 mM potassium cacodylate buffer, pH 7.4. FRET experiments were carried out with a 200 nM oligonucleotide concentration. All labeled DNA oligonucleotides were

supplied by IBA® GmbH. Dual fluorescently labeled DNA oligonucleotides used in these experiments: H-Telo (5′-FAM-GGG TTA GGG TTA GGG TTA GGG-TAMRA-3′), TERRA (5′-FAM-GGG UUA GGG UUA GGG UUA GGG-TAMRA-3′) and ds-DNA (5′-FAM-TAT AGC TAT A-HEG-T ATA GCT ATA-TAMRA-3′) which is a dual-labeled 20-*mer* oligonucleotide comprising a self-complementary sequence with a central polyethylene glycol linker able to fold into a hairpin. The donor fluorophore was 6-carboxyfluorescein (FAM) and the acceptor fluorophore was 6-carboxytetramethylrhodamine (TAMRA). The dual-labeled oligonucleotides were annealed at a concentration of 400 nM by heating at 94 ºC for 10 min followed by slow cooling to rt at a controlled rate of 0.1 ºC/min. 96-well plates were prepared by addition of 50 μl of the annealed DNA solution to each well, followed by 50 μl solution of molecules **1**-**20** at the appropriate concentration. Measurements were made in duplicate with an excitation wavelength of 483 nm and a detection wavelength of 533 nm. Final analysis of the data was carried out using Prism 5 data analysis and graphing software (Prism®).

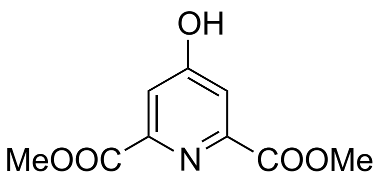

Chelidamic acid dimethyl ester. Chelidamic acid hydrate (2.0 g, 10.16 mmol) was suspended in 20 ml MeOH and $SOCl_2$ 500 μl was added at -10 °C under stirring. The solution was slowly allowed to warm up at room temperature and kept under stirring overnight. After that time the green solution was refluxed 2 h and the solvent removed *in vacuo*. The green oil obtained is re-crystallized from EtOH to afford a withe solid (1.5 g, 7.62 mmol, 70%). ^1H NMR (400 MHz, $CDCl_3$) δ_H 7.39 (2H, *br s*), 3.92 (6H, *s*).

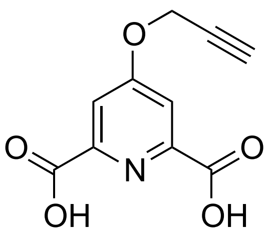

4-(Prop-2-yn-1-yloxy)pyridine-2,6-dicarboxylic acid. Chelidamic acid dimethylester (0.8 g, 3.5 mmol), propargyl alcohol (0.3 ml, 4.6 mmol) and 1.6 g triphenylphosphine polymer bound (3.0 mmol loading/ g) were added to 50 ml freshly distilled THF and cooled to 0 °C. DIAD (0.9 ml, 4.9 mmol) was added dropwise under argon. The mixture was allowed to warm to rt and stirred for 3 d. The solution was filtered and the solvent was removed *in vacuo* and the product purified by column chromatography (50% EtOAc, 50% petroleum ether) to obtain the dimethyl ester of the title compound as a white powder. This compound was dissolved in 50 ml MeOH and deprotected by slowly adding a solution of NaOH (0.3 g, 7.7 mmol) in 50 ml H_2O. The methanol was evaporated *in vacuo* and the remaining suspension re-dissolved by adding H_2O. The solution was acidified with 5% HCOOH (aq.) and extracted with EtOAc. The organic layer was dried over $MgSO_4$, filtered, and the solvent removed *in vacuo* to obtain the title compound as a white powder (0.6 g, 2.7 mmol, 77%). ^{1}H NMR (400 MHz, CD_3OD) δ_H 7.95 (2H, *s*), 5.04 (2H, *d*, *J* 2.5), 3.16 (1H, *t*, *J* 2.5); ^{13}C NMR (100 MHz, CD_3OD) δ_C 167.1, 165.9, 149.5, 114.4, 78.8, 76.8, 56.5; HRMS (ES) calculated for $C_{10}H_8NO_5$ ($[M + H]^+$) m/z: 222.0402, found 222.0398.

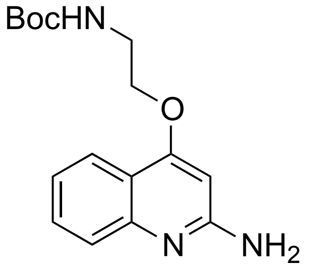

4-(2-*tert*-butoxycarbonylamino-ethoxy)-quinolin-2-ylamine. 2-amino-quinolinone (1.0 g, 6.2 mmol), N-Boc-ethanolamine (1.5 g, 9.3 mmol) and triphenylphosphine (3.3 g, 12.6 mmol) were dissolved in 10 ml freshly distilled THF and cooled to 0 °C. DIAD (1.8 ml, 9.4 mmol) was added dropwise under argon. The mixture was allowed to warm to rt and stirred for 3 d. The solvent was removed

in vacuo and the product purified by column chromatography (90% EtOAc, 10% MeOH) to obtain the title compound as a white powder (1.2 g, 4.0 mmol, 65%). ^{1}H NMR (500 MHz, $CDCl_3$) δ_H 7.97 (1H, *dd*, *J* 8.0, 1.0 Hz), 7.59 (1H, *dd*, *J* 8.5, 1.0 Hz), 7.54 (1H, *ddd*, *J* 8.5, 7.0, 1.0 Hz), 7.23 (1H, *ddd*, *J* 8.0, 7.0, 1.0 Hz), 6.02 (1H, *s*), 5.00 (1H, *br s*), 4.69 (2H, *br s*), 4.16 (2H, *t*, *J* 5.0 Hz), 3.67 (2H, *dd*, *J* 5.0, 5.5), 1.47 (9H, *s*); ^{13}C NMR (125 MHz, $CDCl_3$) δ_C 162.3, 158.0, 155.9, 148.5, 130.3, 125.7, 122.0, 121.6, 117.5, 90.1, 79.8, 67.5, 39.8, 28.4; HRMS (ES) calculated for $C_{16}H_{22}N_3O_3$ ([M + H]$^+$) m/z: 304.1650, found 304.1668.

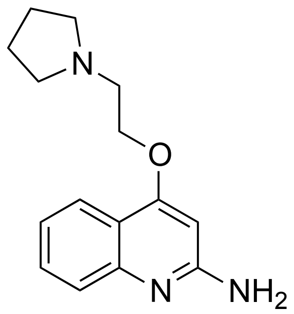

4-(2-Pyrrolidinyl-ethoxy)-quinolin-2-ylamine. 2-Amino-quinolinone (1.5 g, 9.4 mmol), N-(2-hydroxyethyl)-pyrrolidine (1.7 g, 14.5 mmol) and triphenylphosphine (4.9 g, 18.6 mmol) were dissolved in 100 ml of freshly distilled THF and cooled to 0 °C. DIAD (3.8 g, 18.6 mmol) was added dropwise under argon. The mixture was allowed to warm to rt and stirred for 3 d. The solvent was removed *in vacuo* and the product purified by column chromatography (87% EtOAc, 10% MeOH, 3% TEA) to obtain the title compound as a pale yellow powder (1.3 g, 5.0 mmol, 53%). ^{1}H NMR (400 MHz, CD_3OD) δ_H 8.00 (1H, *dd*, *J* 8.0, 1.0 Hz), 7.53 (1H, *dd*, *J* 8.0, 1.5 Hz), 7.48 (1H, *ddd*, *J* 8.0, 6.5, 1.0 Hz), 7.20 (1H, *ddd*, *J* 8.0, 6.5, 1.5 Hz), 6.28 (1H, *s*), 4.33 (2H, *t*, *J* 5.5 Hz), 3.10 (2H, *t*, *J* 5.5 Hz), 2.81-2.73 (4H, *m*), 1.94-1.82 (4H, *m*); ^{13}C NMR (100 MHz, CD_3OD) δ_C 163.0, 160.3, 148.0, 130.2, 124.1, 121.9, 121.6 , 110.8, 90.4, 67.6, 54.8, 54.6, 23.3; HRMS (ES) calculated for $C_{15}H_{20}N_3O$ ([M + H]$^+$) m/z: 258.1606, found 258.1611.

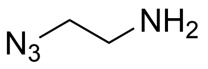

2-Azidoethylamine. 2-chloroethylamine hydrocloride (1g, 8.6 mmol) and NaN_3 (650 mg 9 mmol) were dissolved in water and allowed to stir at 80 °C overnight. After this time the solution was cooled down and basified by addition of a KOH 2 M solution. The product was co-distilled with water and quenched in a HCl solution. The final azide was recovered as a hydrochloride salt after evaporating the solvent under reduced pressure (72% yield). Spectroscopic data were in agreement with previous reports.[1]

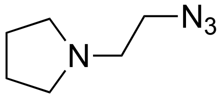

1-(2-Azidoethyl)pyrrolidine. 1-(2-chloroethyl)pyrrolidine hydrochloride (2g, 11.7 mmol) and NaN_3 (1.30g 18 mmol) were dissolved in water and allowed to stir at 80 °C overnight. After this solution was cooled down and basified by addition of a 2M KOH solution, the mixture was extracted 3 times with Et_2O. Organic layers were collected, dried over $MgSO_4$ and the solvent evaporated to afford the product as a brown oil (42 % yield). Spectroscopic data were in agreement with previous reports.[2]

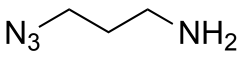

3-Azidopropylamine. 3-bromopropylamine hydrobromide (2g, 8.7 mmol) and NaN_3 (650 mg 9 mmol) were dissolved in water and allowed to stir at 80 °C overnight. After this time the solution was cooled down and basified by addition of a 2M KOH solution. The product was co-distilled with water and quenched in a HCl solution. The final azide was recovered as a hydrochloride salt after evaporating the solvent under reduced pressure (55% yield). Spectroscopic data were in agreement with previous reports.[3]

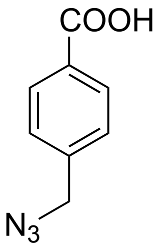

4-Azidomethylbenzoic Acid. Sodium azide (1.3 g, 20 mmol) and 18-crown-6 ether (0.2 mL, 1 mmol) were dissolved in DMSO (4 mL). To the resulting solution was added 4-chloromethyl benzoic acid (1.71g, 10mmol) and the reaction mixture was stirred 12h at 25 °C. The reaction was diluted in EtOAc, washed with 0.1 N HCl (2x), then washed with brine. The organic layer was dried over Na_2SO_4 and concentrated to provide a white solid (1.75 g, quant.). Spectroscopic data were in agreement with previous reports.[4]

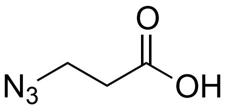

3-Azidopropionic acid. 3-Bromopropionic acid (25 mmol) was dissolved in acetonitrile (40 mL), sodium azide was (50 mmol) added to the solution and the mixture was refluxed for 4h. Acetonitrile was then removed under reduced pressure and the resulting residue was suspended in ethyl acetate (50 mL) and extracted with 0.1 N HCl (3 x 40mL), water (3 x 40 mL) and brine (1 x 30 mL). The organic layer was dried over Na_2SO_4 to afford the 3-azidopropionic acid in 87% yield. Spectroscopic data were in agreement with previous reports.[5]

Pyridostatin precursors 1 and 2. One mole equivalent of **4-(prop-2-yn-1-yloxy)pyridine-2,6-dicarboxylate** was dissolved in DCM (≈ 0.5 M) and 2.2 mole equivalents of 1-chloro-*N*,*N*,2-trimethylpropenyl-amine were added slowly at 0 ºC. The reaction was allowed to stir at rt for 2 h. After total conversion was reached the solution was cooled to 0 ºC and 2.2 mole equivalents of triethylamine were added dropwise. The solution was allowed to warm to rt and stirred for another hour. 2 mole equivalents of 4-(2-*tert*-butoxycarbonylamino-ethoxy)-quinolin-2-ylamine or 4-(2-Pyrrolidinyl-ethoxy)-quinolin-2-ylamine were added to the mixture as DCM

suspensions (≈ 1.0 M) and stirred under argon at rt overnight. Products were precipitated from hot MeCN. Boc-protected compound **1** was dissolved in DCM and deprotected using TFA/DCM (1:2). Both the final compounds were purified by HPLC (gradient: 10% MeCN/90% H_2O, 0.1% TFA to 100% MeCN, 0.1% TFA over 30 min, R_t=15.0-16.5 min).

(1) ^{1}H NMR (400 MHz, CD_3OD) δ_H 8.57 (2H, *d*, *J* 8.0 Hz), 8.27-8.24 (4H, *m*), 8.16 (2H, *d*, *J* 8.0 Hz), 8.08-8.02 (2H, *m*), 7.82-7.76 (2H, *m*), 5.20-5.17 (2H, *m*), 4.89-4.85 (4H, *m*), 3.76-3.70 (4H, *m*), 3.26-3.25 (1H, *m*); ^{13}C NMR (100 MHz, CD_3OD) δ_C 167.7, 167.2, 163.9, 151.3, 149.5, 139.1, 134.1, 127.5, 123.6, 121.4, 118.8, 114.2, 94.6, 78.6, 76.4, 67.4, 56.8, 38.6; HRMS (ES) calculated for $C_{32}H_{30}N_7O_5$ ([M + H]$^+$) m/z: 592.2284, found 592.2297.

(2) ^{1}H NMR (400 MHz, CD_3OD) δ_H 8.35 (2H, *d*, *J* 8.0 Hz), 8.10-8.20 (4H, *m*), 7.91 (2H, *d*, *J* 8.0 Hz), 7.88-7.82 (2H, *m*), 7.62-7.56 (2H, *m*), 5.10-5 (2H, *s*), 4.70 (4H, *t,* 3.2 Hz), 3.90 (4H, *t,* 2.2 Hz), 3.80-3.65 (8H, *m*) 2.4-2.1 (16H, *m*); ^{13}C NMR (100 MHz, CD_3OD) δ_C 168.6, 164.0, 153.1, 152.0, 135.0, 132.4, 128.8, 127.7, 126.6, 123.2, 120.5, 114.0, 96.1, 79.0, 77.9, 65.7, 57.7, 56.2, 54.9 23.9; HRMS (ES) calculated for $C_{40}H_{42}N_7O_5$ ([M + H]$^+$) m/z: 700.3247, found 700.3288.

General Method 3 for 1,4- adducts synthesis *via* click chemistry. One mole equivalent of compound **1** or **2** (20 mg) was dissolved in a 1:2 mixture of ^{t}BuOH/H_2O (1-3 ml). Copper(II) sulfate pentahydrate (100 μL, 100 mM) and sodium ascorbate (300 μl, 100 mM) were added and the solution stirred for 10 min. The respective azide (1 mole equivalent) was added and the solution was allowed to stir overnight

under argon. The solvent was removed under reduced pressure and the products purified by HPLC (gradient: 10% MeCN/90% H_2O, 0.1% TFA to 100% MeCN, 0.1% TFA over 30 min).

2. NMR and HRMS spectral data

(9) ^{1}H NMR (500 MHz, CD_3OD) δ_H 8.60 (2H, *d*, *J* 8.5 Hz), 8.25 (1H, *s*), 8.21 (2H, *s*), 8.15 (2H, *s*), 8.04 (2H, *d*, *J* 8.5 Hz), 7.93-7.89 (2H, *m*), 7.68-7.63 (2H, *m*), 5.55 (2H, *s*), 4.74 (4H, *t*, *J* 5.0 Hz), 4.40 (2H, *t, J* 4.0 Hz), 3.66 (4H, *t*, *J* 5.0 Hz), 3.0 (2H, *t, J* 4.0 Hz); ^{13}C NMR (125 MHz, CD_3OD) δ_C 169.3, 166.5, 164.0, 153.3, 151.1, 144.4, 143.0, 134.3, 127.6, 126.3, 124.5, 124.0, 119.8, 114.0, 96.1, 67.4, 63.0, 54.8, 38.9, 38.0; HRMS (ES) calculated for $C_{34}H_{36}N_{11}O_5$ ($[M + H]^+$) m/z: 678.2901, found 678.2913.

(10) ^{1}H NMR (500 MHz, CD_3OD) δ_H 8.44-8.42 (3H, *m*), 8.21 (2H, *s*), 8.16 (2H, *s*), 8.00 (2H, *br d*, *J* 8.5 Hz), 7.89-7.86 (2H, *m*), 7.64-7.61 (2H, *m*), 5.65-5.63 (1H, *m*), 5.56 (2H, *s*), 4.71-4.70 (4H, *m*), 3.91-3.84 (2H, *m*), 3.65-3.55 (4H, *m*), 3.43-3.37 (4H, *m*); ^{13}C NMR (125 MHz, CD_3OD) δ_C 165.9, 164.1, 152.1, 151.3, 145.0, 142.2, 133.6, 127.2, 125.5, 125.4, 123.9, 120.3, 114.3, 95.4, 89.7, 81.2, 78.5, 74.1, 70.9, 67.2, 63.4, 62.4, 54.8, 39.8; = +21.0° (c 100 μM, H_2O); HRMS (ES) calculated for $C_{38}H_{41}N_{10}O_{10}$ ($[M + H]^+$) m/z: 797.3038, found 797.3001.

(11) ^{1}H NMR (500 MHz, CD_3OD) δ_H 8.47 (2H, *d*, *J* 8.5 Hz), 8.25 (1H, *s*), 8.21 (2H, *s*), 8.15 (2H, *s*), 8.04 (2H, *d*, *J* 8.5 Hz), 7.93-7.89 (2H, *m*), 7.68-7.63 (2H, *m*), 5.55 (2H, *s*), 4.74 (4H, *t*, *J* 5.0 Hz), 4.61-4.55 (2H, *m*), 3.66 (4H, *t*, *J* 5.0 Hz), 3.04-2.97 (2H, *m*), 2.33-2.25 (2H, *m*); ^{13}C NMR (125 MHz, CD_3OD) δ_C 169.4, 166.5, 164.2, 152.5, 151.2, 144.0, 143.5, 134.0, 127.5, 126.3, 124.8, 124.1, 120.2, 114.3, 95.3, 67.5, 63.5, 54.8, 39.8, 38.1, 29.1; HRMS (ES) calculated for $C_{35}H_{38}N_{11}O_5$ ($[M + H]^+$) m/z: 692.3057, found 692.3051.

(12) ^{1}H NMR (500 MHz, CD_3OD) δ_H 8.51 (2H, *d*, *J* 8.5 Hz), 8.30 (1H, *s*), 8.15 (2H, *s*), 8.10 (2H, *s*), 8.00 (2H, *d*, *J* 8.5 Hz), 7.85-7.80 (2H, *m*), 7.65-7.60 (2H, *m*), 5.55 (2H, *s*), 4.70 (2H, *t*, *J* 4.5 Hz), 4.61-4.55 (4H, *m*), 3.90 (2H, *t*, *J* 4.5 Hz), 3.70-3.60 (4H, *m*), 2.33-2.25 (8H, *m*); ^{13}C NMR (125 MHz, CD_3OD) δ_C 169.4, 165.6, 164.1, 152.8, 151.6, 144.0, 143.5, 133.3, 127.1, 126.9, 126.0, 123.8, 120.4, 114.1, 95.5, 67.1, 63.4, 55.7, 54.8, 47.3, 39.9, 23.9; HRMS (ES) calculated for $C_{38}H_{42}N_{11}O_5$ ($[M + H]^+$) m/z: 732.3365, found 732.3345.

(13) ^{1}H NMR (500 MHz, CD_3OD) δ_H 8.50 (2H, *d*, *J* 8.5 Hz), 8.15 (1H, *s*), 8.00 (2H, *s*), 7.90 (2H, *s*), 7.75 (2H, *d*, *J* 8.5 Hz), 7.40-7.30 (2H, *m*), 7.20-7.10 (2H, *m*), 5.50 (2H, *s*), 4.50 (4H, *t*, *J* 5.0 Hz), 4.10 (2H, *t, J* 4.5 Hz), 3.50 (4H, *t*, *J* 5.0 Hz), 3.10 (2H, *t, J* 4.0 Hz); ^{13}C NMR (125 MHz, CD_3OD) δ_C 172.5, 168.8, 167.2, 164.1, 153.3, 151.5, 146.1, 143.5, 133.4, 128.3, 126.0, 124.9, 124.5, 120.1, 115.1, 94.8, 65.6, 63.9, 55.5, 42.4, 38.9; HRMS (ES) calculated for $C_{35}H_{35}N_{10}O_7$ ($[M + H]^+$) m/z: 707.2690, found 707.2659.

(14) ^{1}H NMR (400 MHz, CD_3OD) δ_H 8.49 (2H, *d*, *J* 8.5 Hz), 8.30 (1H, *s*), 8.18 (2H, *s*), 8.13 (2H, *s*), 8.07-7.98 (4H, *m*), 7.97-7.90 (2H, *m*), 7.71-7.64 (2H, *m*), 7.42 (2H, *d*, *J* 8.5 Hz), 5.76 (2H, *s*), 5.59 (2H, *s*), 4.79 (4H, *t*, *J* 5.0 Hz), 3.72 (4H, *t*, *J* 5.0 Hz); ^{13}C NMR (100 MHz, CD_3OD) δ_C 168.4, 168.0, 166.0, 163.2, 151.3, 149.9, 143.9, 143.3, 142.6, 142.2, 140.8, 133.2, 130.3, 128.0, 126.3, 125.4, 123.2, 119.2, 113.7, 94.2, 66.8, 62.6, 53.4, 38.8; HRMS (ES) calculated for $C_{40}H_{37}N_{10}O_7$ ($[M + H]^+$) m/z: 769.2847, found 769.2841.

(15) ^{1}H NMR (400 MHz, DMSO) δ_H 8.45 (2H, *m*), 8.30 (1H, *s*), 8.18 (2H, *s*), 8.10-8.00 (4H, *m*), 7.90-7.80 (2H, *m*), 7.60-7.55 (2H, *m*), 5.60 (2H, *s*), 5.00 (4H, *t*, *J* 5.0 Hz), 4.80-4.75 (2H, *m*), 4.05 (2H, *m*), 3.90 (4H, *t*, *J* 5.0 Hz), 3.40 (8H, *bs*), 2.20 (8H, *bs*); ^{13}C NMR (100 MHz, CD_3OD) δ_C 168.9, 165.0, 163.0, 161.1, 152.3, 148.9, 143.9, 143.3, 133.5, 126.9, 126.0, 123.1, 122.7, 113.7, 94.3, 65.9, 62.5, 55.0, 53.9, 53.5, 46.3, 23.0; HRMS (ES) calculated for $C_{42}H_{48}N_{11}O_5$ ($[M + H]^+$) m/z: 786.3840, found 786.3801.

(16) ^{1}H NMR (400 MHz, CD_3OD) δ_H 8.50 (1H, *s*), 8.35 (2H, *d*, *J* 8.0 Hz), 8.11 (2H, *s*), 7.95 (2H, *s*), 7.75 (2H, *d, J* 8.0 Hz), 7.65 (2H, *m*), 7.55 (2H, *m*), 5.70 (1H, *d, J* 10Hz), 5.50 (2H, *s*), 4.79-4.61 (4H, *m*), 4.10-3.99 (4H, *m*), 3.90-3.85 (2H, *m*), 3.75-3.70 (1H, *m*), 3.65-3.60 (2H, *m*), 3.55-3.50 (2H, *m*), 3.40 (8H, *bs*), 2.20 (8H, *bs*) ; ^{13}C NMR (100 MHz, CD_3OD) δ_C 168.9, 163.8, 162.5, 151.0, 150.0, 148.4, 142.6, 132.9, 126.5, 124.6, 123.4, 122.9, 113.9, 113.5, 94.1, 88.7, 80.2, 77.5, 73.1, 69.9, 65.5, 62.5, 61.4, 55.1, 53.5, 23.0; HRMS (ES) calculated for $C_{46}H_{53}N_{10}O_{10}$ ($[M + H]^+$) m/z: 905.3941, found 905.3916.

(17) ^{1}H NMR (400 MHz, CD_3OD) δ_H 8.50 (1H, *s*), 8.35 (2H, *d*, *J* 8.0 Hz), 8.11 (2H, *s*), 7.95 (2H, *s*), 7.75 (2H, *d, J* 8.0 Hz), 7.65 (2H, *m*), 7.55 (2H, *m*), 5.55 (2H, *s*), 4.74 (4H, *t*, *J* 5.0 Hz), 4.61-4.55 (2H, *m*), 3.66 (4H, *t*, *J* 5.0 Hz), 3.45-3.30 (8H, *m*) 3.04-2.97 (2H, *m*), 2.33-2.25 (10H, *m*); ^{13}C NMR (100 MHz, CD_3OD) δ_C 168.1, 164.1, 162.3, 151.5, 150.0, 148.2, 142.5, 136.1, 126.9, 124.6, 123.9, 122.1, 114.0, 113.9, 96.1, 71.2, 66.5, 61.7, 61.4, 55.0, 54.5, 24.2, 23.0; HRMS (ES) calculated for $C_{43}H_{50}N_{11}O_5$ ($[M + H]^+$) m/z: 800.3996, found 800.3959.

(18) ^{1}H NMR (400 MHz, CD_3OD) δ_H 8.50 (1H, *s*) 8.40 (2H, *d*, *J* 8.5 Hz), 8.22 (2H, *s*), 8.20 (2H, *s*), 8.12 (2H, *d*, *J* 8.5 Hz), 8.02-7.96 (2H, *m*), 7.75-7.70 (2H, *m*), 5.50 (2H, *s*) 4.95-4.90 (4H, *m*), 3.78-3.74 (2H, *m*), 4.05-3.76 (12H, *m*), 3.50-3.35 (6H, *m*), 2.34-2.07 (12H, *m*); ^{13}C NMR (125 MHz, CD_3OD) δ_C 165.9, 163.1, 155.6, 151.3, 150.1, 148.2 142.0, 133.3, 126.8, 123.1, 119.1, 118.5, 115.5, 113.4, 94.4, 65.8, 64.7, 55.1, 55.0, 54.9, 53.6, 47.3, 23.0, 22.9; HRMS (ES) calculated for $C_{46}H_{54}N_{11}O_5$ ([M + H]$^+$) m/z: 840.4309, found 840.4292.

(19) ^{1}H NMR (400 MHz, CD_3OD) δ_H 8.40 (2H, *d*, *J* 8.0 Hz), 8.30 (1H, *s*), 8.15 (2H, *s*), 8.00 (2H, *s*), 7.90 (2H, *d, J* 8.0 Hz), 7.80-7.75 (2H, *m*), 7.71-7.64 (2H, *m*), 7.60-7.55 (2H, *m*), 5.60 (2H, *s*), 4.80 (2H, *t, J* 5.5 Hz), 4.10-4.05 (4H, *m*), 3.90-3.75 (4H, *m*), 3.60-3.40 (4H, *m*), 3.30-3.25 (4H, *m*), 3.05 (2H, *t, J* 5.5 Hz), 2.20-2.10 (8H, *m*) ^{13}C NMR (100 MHz, CD_3OD) δ_C 173.1, 168.8, 165.8, 163.0, 153.2, 150.0, 149.9, 133.9, 133.6, 126.7, 125.7, 123.3, 122.9, 113.9, 94.2, 65.7, 62.5, 55.1, 53.6, 46.2, 34.0, 23.1; HRMS (ES) calculated for $C_{43}H_{47}N_{10}O_7$ ([M + H]$^+$) m/z: 815.3629, found 815.3626.

(20) ^{1}H NMR (400 MHz, CD_3OD) δ_H 8.55 (2H, *d*, *J* 8.5 Hz), 8.40 (1H, *s*), 8.20 (2H, *s*), 8.10 (2H, *s*), 8.07-7.98 (4H, *m*), 7.95-7.90 (2H, *m*), 7.70-7.60 (2H, *m*), 7.35 (2H, *d*, *J* 8.5 Hz), 5.76 (2H, *s*), 5.59 (2H, *s*), 4.79 (4H, *t*, *J* 5.0 Hz), 3.72 (4H, *t*, *J* 5.0 Hz), 3.44-3.35 (8H, *m*), 2.20-2.10 (8H, *m*); ^{13}C NMR (100 MHz, CD_3OD) δ_C 169.1, 168.4, 165.0, 160.1, 155.2, 151.9, 147.6, 144.6, 143.6, 142.2, 141.1, 133.2, 130.0, 127.6, 126.0, 123.4, 121.2, 114.1, 113.9, 96.1, 65.8, 64.6, 53.4, 47.3, 38.8, 23.7; HRMS (ES) calculated for $C_{48}H_{49}N_{10}O_7$ ([M + H]$^+$) m/z: 877.3768, found 877.3786.

3. Molecular structures of compounds 9-20

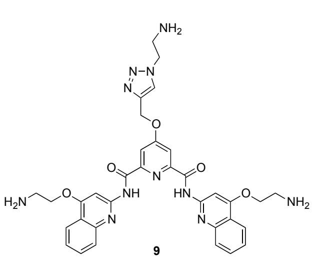

9

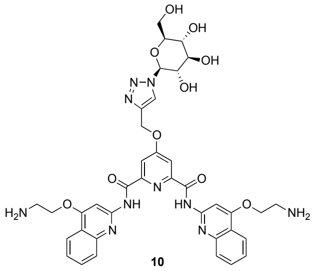

10

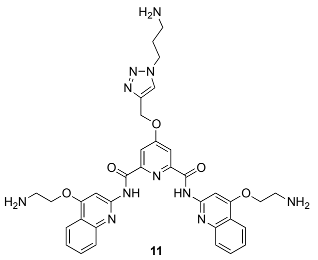

11

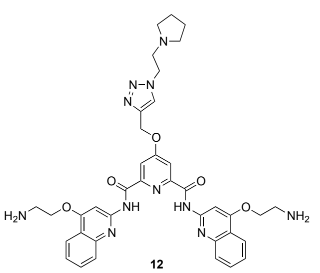

12

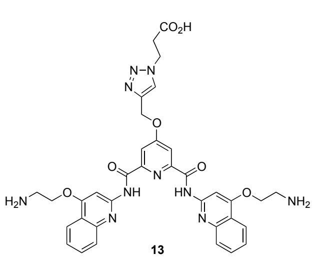

13

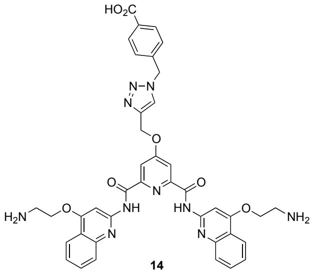

14

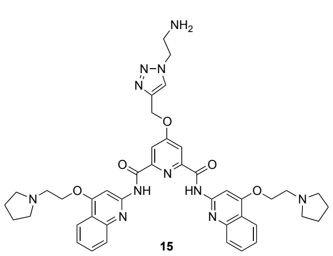

15

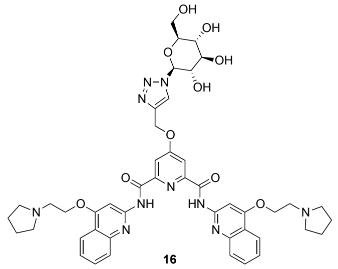

16

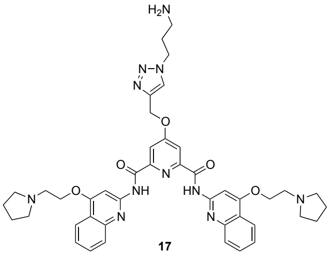

17

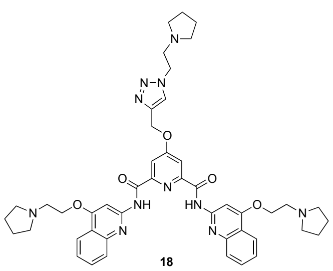

18

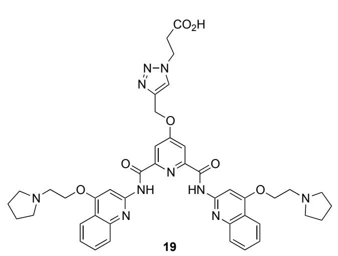

19

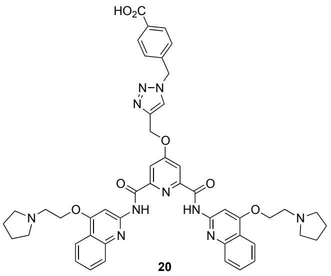

20

4. Click chemistry procedures

DNA sequences used in the click selection experiments are the following: H-Telo; 5′-AGGGTTAGGGTTAGGGTTAGGGT-3′, TERRA; 5′-AGGGUUAGGGUUAGGGUUAGGGU-3′ and ds-DNA; 5′-CAATCGGATCGAATTCGATCCGATTG-3′ (self complementary sequence).

General procedure for Cu free *in situ* click chemistry: In a 1.5 ml eppendorf, 5 μl of oligonucleotide solution at 100 μM (ds-DNA or H-Telo), or water, was added to 1 μL Tris·HCl buffer (200 mM, pH 7.4) and 1 μL of KCl (2M). Solutions were heated at 95 °C for 10 min and kept at 4 °C overnight. Then, 0.5 μL of **1** (1mM) and 0.5 μL of **2** (1mM) were added to the mixture, followed by the addition of 2 μL of each azide **3**-**8** (10mM). The final solution (20 μL) was stirred at r.t. for 6 days before being quenched with 40 μL of a 20% TFA aq. solution and evaporated by means of speed vacuum. The solid obtained was solubilized in 20 μL of water and analyzed by LC-MS.

General procedure for Cu-catalyzed click chemistry: In a 1.5 ml eppendorf, 0.25 μl of oligonucleotide solution at 100 μM (ds-DNA, H-Telo or TERRA), or water, was added to 1 μL Tris·HCl buffer (200 mM, pH 7.4) and 1 μL of KCl (2 M). Solutions were heated at 95 °C for 10 min and kept at 4 °C overnight. Then, 0.5 μL of **1** (1 mM) and 0.5 μL of **2** (1 mM) were added to the mixture, followed by the addition of 2 μL of azides **3**,**5** and **6** (10 mM), 0.5 μL of azides **4** and **7** (10 mM) and 0.25 μL of azide **8** (10 mM). 11.5 μL of water was added followed by 0.5 μl of $CuSO_4 \cdot 5H_2O$ (10 mM) and 0.5 μl of aq. sodium ascorbate (10 mM). The final solution (20 μL) was stirred at r.t. for 3h before being quenched with 40 μL of a 20% TFA aq. solution and

evaporated by means of speed vacuum. The solid obtained was solubilized in 20 μL of water and analyzed by LC-MS.

5. HPLC method and chromatograms

5 μl of a solution obtained according to the methodology described in the experimental were injected in the LCMS: LC Ultimate 3000 Dionex MS Ion Trap AmaZon X Bruker (HyStar software). The system was equipped with an Eclipse Agilent column 3.5 μm XDB-C18 3.0 x 75 mm. Flow rate 1 ml/min CH_3CN/H_2O (0.1% TFA) 15:85 to 25:75 over 30 mins 25:75 to 32:68 next 20 minutes.

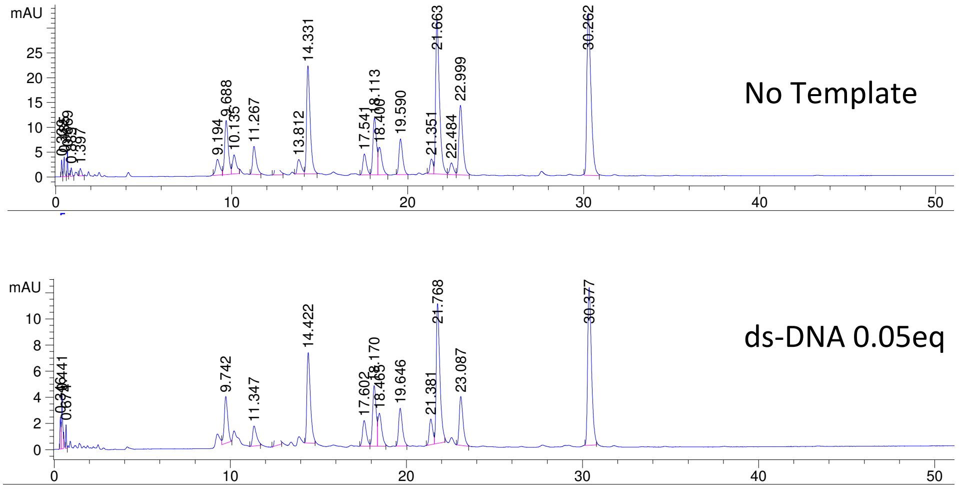

Figure S1: Chromatographic traces for the Cu catalyzed 1,3-dipolar cycloaddition obtained in the presence or in the absence of 0.05 eq of ds-DNA. Negligible changes in the product distribution are detected under these conditions.

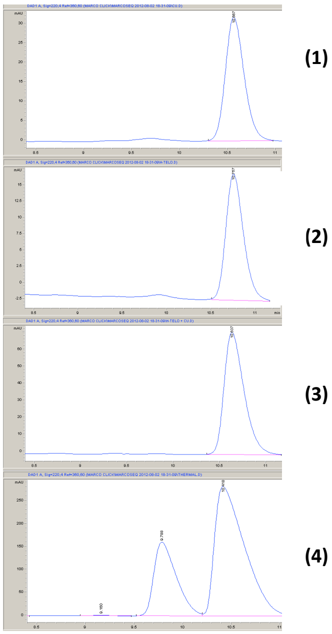

Figure S2: Chromatographic traces for the 1,3-dipolar cycloaddition between 1 and 4 obtained in: (1) the Cu catalyzed reaction leading to the 1,4- regiosomer; (2) the H-Telo catalyzed reaction; (3) the co-injection of (1) and (2); (4) the reaction carried out at 70 °C for 12 hours, leading to a mixture of both the 1,4- and 1,5- regioisomers.

6. Products distribution for Cu catalyzed click reactions

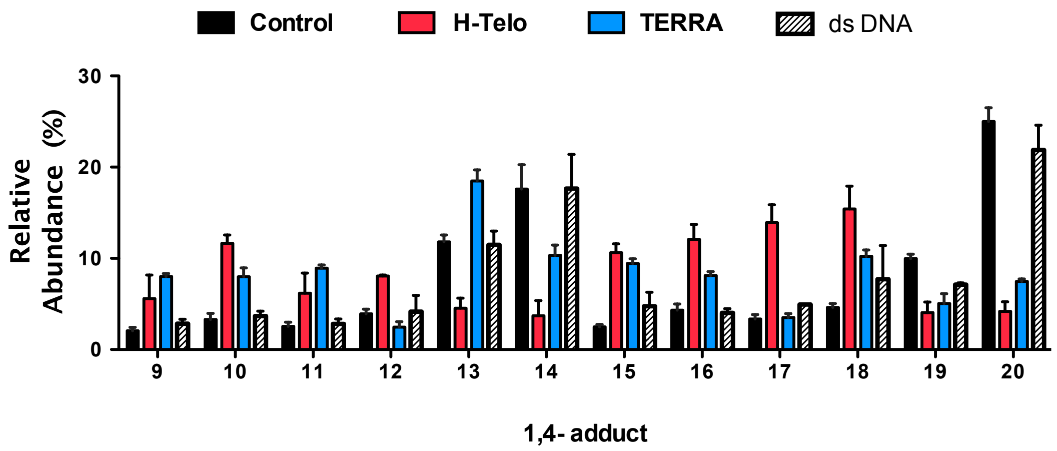

Figure S3: Histogram showing the relative abundance of each product obtained from Cu (Control), Cu/H-Telo, Cu/TERRA and Cu/ds-DNA catalyzed reactions. Data was plotted for the surface area measured under each individual chromatogram signal.

7. FRET-melting curves

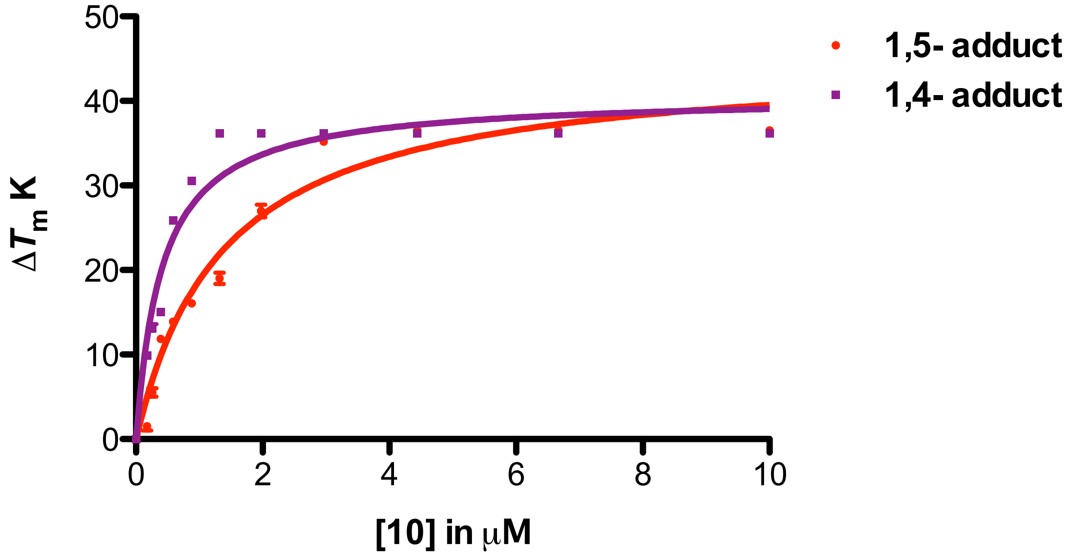

Figure S4: FRET-melting curves obtained by titrating a 200 nM solution of a dual labeled H-Telo with both regioisomers of **10** in a potassium cacodylate-containing buffer (60 mM pH 7.4).

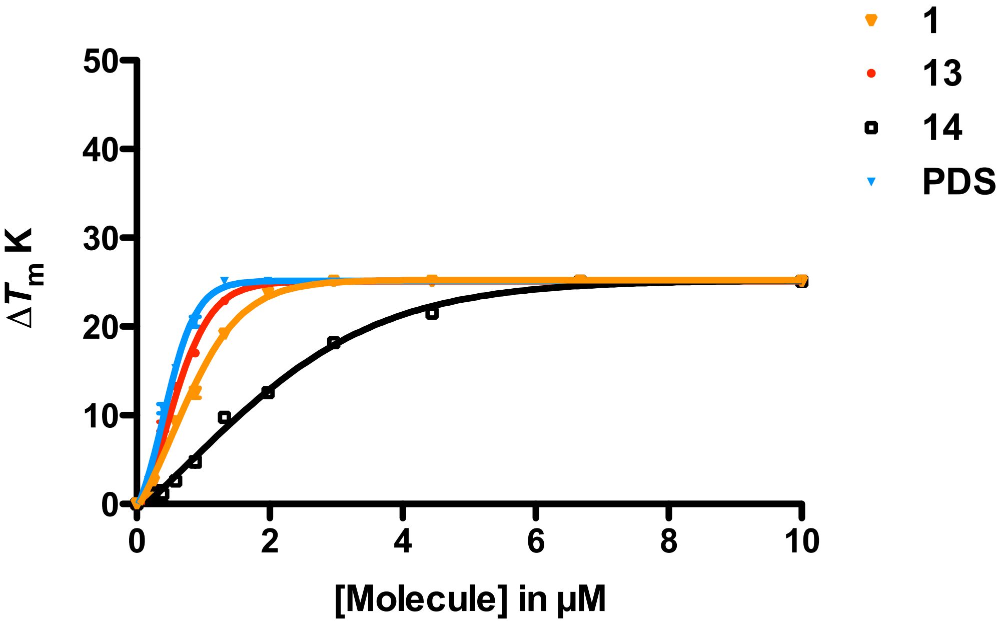

Figure S5: FRET-melting curves obtained by titrating a 200 nM solution of a dual labeled TERRA with **1**, **13**, **14** and PDS in a potassium cacodylate-containing buffer (60 mM pH 7.4).

8. TRF1 immunostaining

Immunofluorescence analysis. SV40-transformed MRC-5 cells were cultured in standard media [DMEM (Dulbecco's Modified Eagle Medium, Celbio), 1% L-glutamine, 10% FBS (fetal bovine serum)], at 37 °C with 5% CO_2. Cells were treated for 24h with 2 μM compound unless stated otherwise. For immunofluorescence analysis, cells were fixed 20 min in 2% paraformaldehyde/PBS, permeabilized 10 min with 0.1% Triton X/PBS and blocked for 1h with 2% Marvel™/PBS (blocking buffer). Slides were incubated with an anti-TRF1 antibody (ab10579, Abcam, UK) diluted 1/100 in blocking buffer for 1h at 37 °C in humid chamber. Slides were then washed 3 times with 0.1% Tween 20/PBS, and incubated as described above with an anti-mouse Alexa 488-conjugated secondary antibody (A11029, Invitrogen) diluted 1/500 in blocking buffer. Slides were washed as described above and mounted with Prolong Gold with DAPI (Invitrogen). Images were taken with an Axioskop 2 *plus*

microscope (Zeiss) and analysed with Volocity software (Perkin Elmer) for foci distribution. Frequency distribution graphs were plotted with GraphPad Prism software (USA). TRF1 *foci* counts were performed on > 100 cells for each treatment.

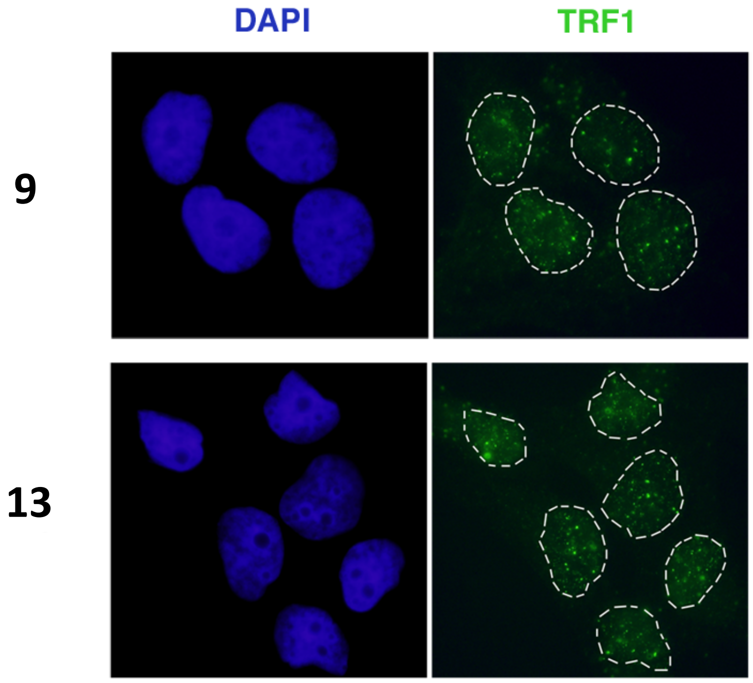

Figure S6: Fluorescent microscopy images of MRC5-SV40 cells treated with 2 µM compound for 24h. Cells were stained with DAPI and anti-TRF1 antibody.

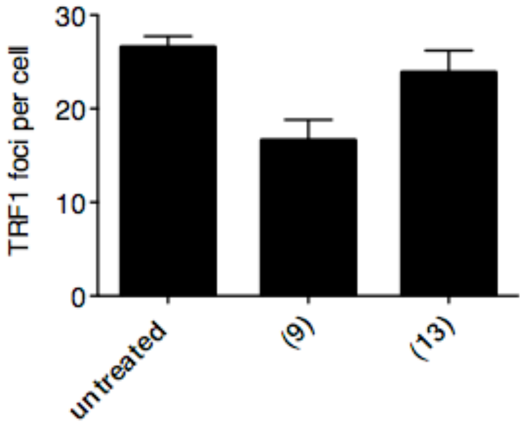

Figure S7: Number of TRF1 *foci* per cell upon treatment with **9** and **13** (2 µM, 24h).

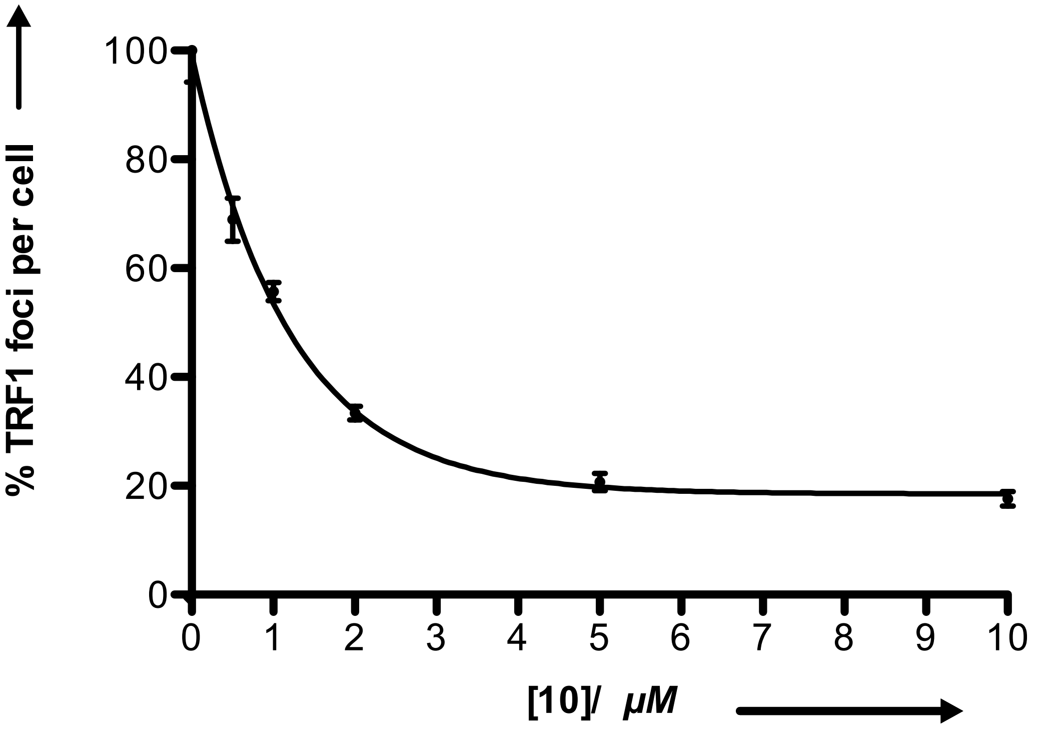

Figure S8: Dose-response of TRF1 displacement with **10**.

9. References

[1] I. A. Inverarity, R. F. Viguier, P. Cohen, A. N. Hulme, *Bioconj. Chem.* **2007**, *18*, 1593-1603.

[2] J. Dash, Z. A. Waller, G. D. Pantos, S. Balasubramanian, *Chemistry* **2011**, *17*, 4571-4581.

[3] F. Landi, C. M. Johansson, D. J. Campopiano, A. N. Hulme, *Org. Biomol. Chem.* **2010**, *8*, 56-59.

[4] K. Sakurai, T. M. Snyder, D. R. Liu, *J. Am. Chem. Soc.* **2005**, *127*, 1660-1661.

[5] L. Le Corre, A. L. Girard, J. Aubertin, F. Radvanyi, C. Benoist-Lasselin, A. Jonquoy, E. Mugniery, L. Legeai-Mallet, P. Busca, Y. Le Merrer, *Org. Biomol. Chem.* **2010**, *8*, 2164-2173.
